# Controlled Human Malaria Infection Induces Long-Term Functional Changes in Monocytes

**DOI:** 10.3389/fmolb.2020.604553

**Published:** 2020-11-26

**Authors:** Jona Walk, Farid Keramati, L. Charlotte J. de Bree, Rob J. W. Arts, Bas Blok, Mihai G. Netea, Hendrik G. Stunnenberg, Robert W. Sauerwein

**Affiliations:** ^1^Department of Medical Microbiology and Radboud Center for Infectious Diseases, Radboud University Medical Center, Nijmegen, Netherlands; ^2^Department of Internal Medicine and Radboud Center for Infectious Diseases, Radboud University Medical Center, Nijmegen, Netherlands; ^3^Department of Molecular Biology, Faculty of Science, Radboud University, Nijmegen, Netherlands; ^4^Research Center for Vitamins and Vaccines, Bandim Health Project, Statens Serum Institut, Copenhagen, Denmark; ^5^Odense Patient Data Explorative Network, University of Southern Denmark/Odense University Hospital, Odense, Denmark; ^6^Department for Immunology and Metabolism, Life and Medical Sciences Institute (LIMES), University of Bonn, Bonn, Germany

**Keywords:** malaria, trained immunity, epigenetics, RNA-seq, ChIP-seq, CHMI, innate immunity

## Abstract

Innate immune memory responses (also termed “*trained immunity*”) have been described in monocytes after BCG vaccination and after stimulation *in vitro* with microbial and endogenous ligands such as LPS, β-glucan, oxidized LDL, and monosodium urate crystals. However, whether clinical infections are also capable of inducing a trained immunity phenotype remained uncertain. We evaluated whether *Plasmodium falciparum* infection can induce innate immune memory by measuring monocyte-derived cytokine production from five volunteers undergoing Controlled Human Malaria Infection. Monocyte responses followed a biphasic pattern: during acute infection, monocytes produced lower amounts of inflammatory cytokines upon secondary stimulation, but 36 days after malaria infection they produced significantly more IL-6 and TNF-α in response to various stimuli. Furthermore, transcriptomic and epigenomic data analysis revealed a clear reprogramming of monocytes at both timepoints, with long-term changes of H3K4me3 at the promoter regions of inflammatory genes that remain present for several weeks after parasite clearance. These findings demonstrate an epigenetic basis of trained immunity induced by human malaria *in vivo*.

## Introduction

Throughout our life, the immune system continually encounters a wide array of microorganisms and other danger signals. Until recently, exposure to microbial ligands or infectious agents was thought to induce immune memory exclusively in T and B lymphocytes. However, epigenetic reprogramming of monocytes (Cheng et al., 2014; Saeed et al., 2014; Arts et al., 2016) can result in long-lasting changes in the innate immune compartment after exposure to various microorganisms (Kleinnijenhuis et al., 2012; Quintin et al., 2012). Epigenetic reprogramming underlies these effects (Cheng et al., 2014; Saeed et al., 2014; Arts et al., 2016). This can cause either decreased pro-inflammatory cytokine production (e.g., after LPS exposure), a process known as “tolerance,” or increased cytokine production seen after exposure to BCG (Arts et al., 2018), or β-glucan (Saeed et al., 2014), known as “trained immunity” (reviewed in Dominguez-Andres and Netea, 2018). Trained immunity is thought to underlie some clinical observations of heterologous protective effects after vaccination, including the observed reduction in all cause infant mortality after BCG and measles vaccination (de Bree et al., 2018).

Acute malaria infection is also associated with activation of monocytes through TLR-dependent responses (McCall et al., 2007; Ataide et al., 2014). Interestingly, it was demonstrated recently that *in vitro* exposure to *Plasmodium falciparum* or the parasite pigment haemozoin increased monocyte cytokine responses to re-stimulation with a TLR2 agonist (Schrum et al., 2018), leading many to theorize that malaria can induce trained immunity in humans (Ortega-Pajares and Rogerson, 2018; Dobbs et al., 2020). However, whether physiological concentrations of *P. falciparum* induce trained immunity *in vivo* has not been demonstrated.

Here we determined monocyte responses in five healthy volunteers undergoing controlled human malaria infection (Sauerwein et al., 2011), and demonstrate that even at very low parasite densities *P. falciparum* induces functional changes in monocytes lasting at least one month, suggestive of trained immunity. Transcriptomic and epigenomic analyses reveal that these changes occur on a genome-wide scale at both the RNA level and H3K4me3 histone marks.

## Results

### Controlled *in vivo* Malaria Infection Induces Trained Immunity in Monocytes

**FIGURE 1:**
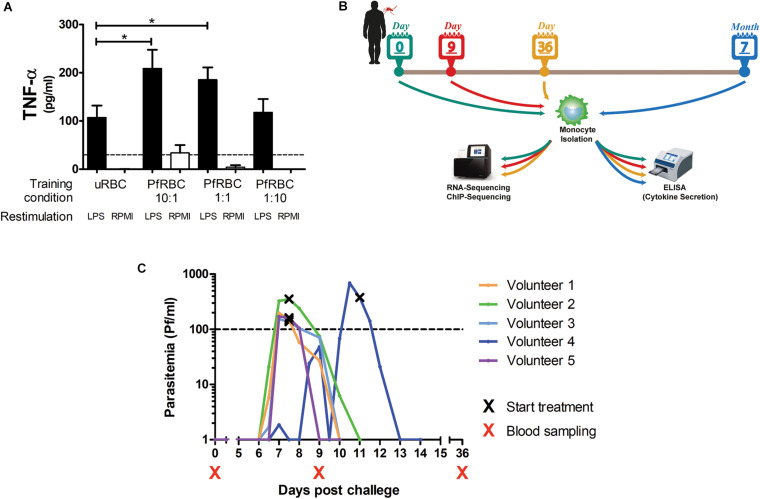
Controlled Human Malaria Infection induces lasting changes in monocytes. **(A)** Adherent monocytes from 6 healthy donors were seeded at 0.1 × 10^6^/well and pre-incubated with uninfected red blood (uRBC) cells at a concentration or 1× 10^6^/well, or *P. falciparum* infected red blood cells (PfRBC) at a concentration of 1 × 10^6^/well, 0.1 × 10^6^/well or 0.01 × 10^6^/well for 24 h. After 5 days resting in normal medium, monocytes were restimulated with 10 ng/mL LPS (black bars) or RPMI as a negative control (white bars) for 24 h. The graph shows TNF-α concentrations measured in supernatants by ELISA. *P*-values are the result of Wilcoxon matched-pairs signed rank test, *p < 0.05. **(B)** Five healthy volunteers were infected with *P. falciparum* by mosquito bite challenge, blood was sampled at baseline (before infection), and 9 and 36 days, and 7 months after infection. **(C)** The graph shows the parasitemia as determined by twice daily qPCR starting on day 6 after challenge for each individual volunteer. All volunteers were treated when parasitemia reached 100Pf/mL. Four volunteers were treated on day 7 after challenge and one volunteer on day 11 after challenge. *p < 0.05.

Monocytes isolated from healthy donors were incubated with different concentrations of *P. falciparum*-infected red blood cells for 24 h, allowed to rest in culture medium for 5 days, and then restimulated with LPS. There was a dose-dependent increase in TNF-α production upon LPS stimulation when monocytes had been pre-incubated with infected red blood cells (Figure 1A).

**FIGURE 2:**
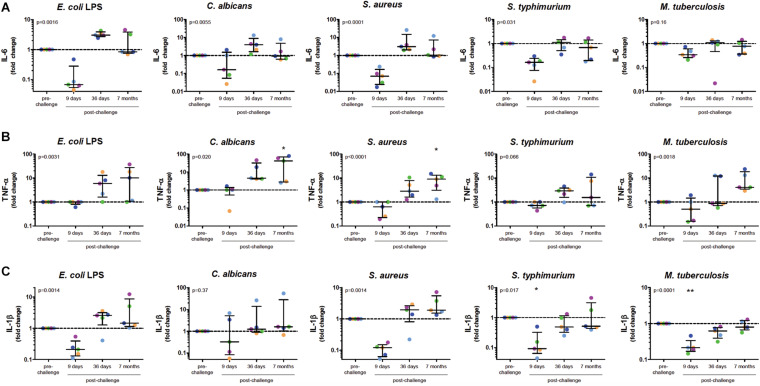
Monocytes after Controlled Human Malaria Infection Show Trained Innate Immune Memory Phenotype. Monocytes at baseline (before infection), 9 days after CHMI challenge, 36 days after challenge and 7 months after challenge, were isolated from PBMC and stimulated for 24 h with LPS, *C. albicans*, *S. aureus*, *S. typhimurium*, or *M. tuberculosis*. Graphs show supernatant IL-6 **(A)**, TNF-α **(B)**, and IL-1β **(C)** concentrations determined by ELISA as fold change over the baseline measurements. Dots represent individual volunteers (colors represent each volunteer). *P*-values shown are the results of Friedman Repeated Measures ANOVA. If time points differed significantly from baseline after correction for multiple testing using Dunn’s Multiple Comparison Test this is indicated above the appropriate column. *p < 0.05, **p < 0.01.

To investigate whether *in vivo* infection also induced this training effect, five volunteers were infected with *P. falciparum* sporozoites in a controlled manner (Figure 1B). All five volunteers developed parasitemia detectable by qPCR after infection (Figure 1C). Baseline characteristics and parasitological data for each volunteer are shown in Supplementary Table 1. CD14+ cells were isolated pre-infection (baseline), on day 9 after infection, day 36, and 7 months after infection, and treated with different stimuli. During parasitemia (day 9) monocytes produced less IL-6 and IL-1β compared to baseline in response to all tested stimuli (Figures 2A–C). In contrast, 36 days after infection, more than 3 weeks after parasite clearance, IL-6 and TNF-α production was significantly increased upon stimulation with LPS, *C. albicans* and *S. aureus* compared to baseline (Figures 2A,B). 7 months after infection, IL-6 production returned to baseline, though TNF-α and IL-1β (the latter in case of LPS and *S. aureus* stimulations) production remained increased compared to baseline. Monocytes from most donors did not produce detectable amounts of IL-10 upon stimulation at any time point (data not shown).

### Controlled Malaria Infection Induces Genome-Wide Transcriptomic and Epigenomic Reprogramming in Monocytes

**FIGURE 3:**
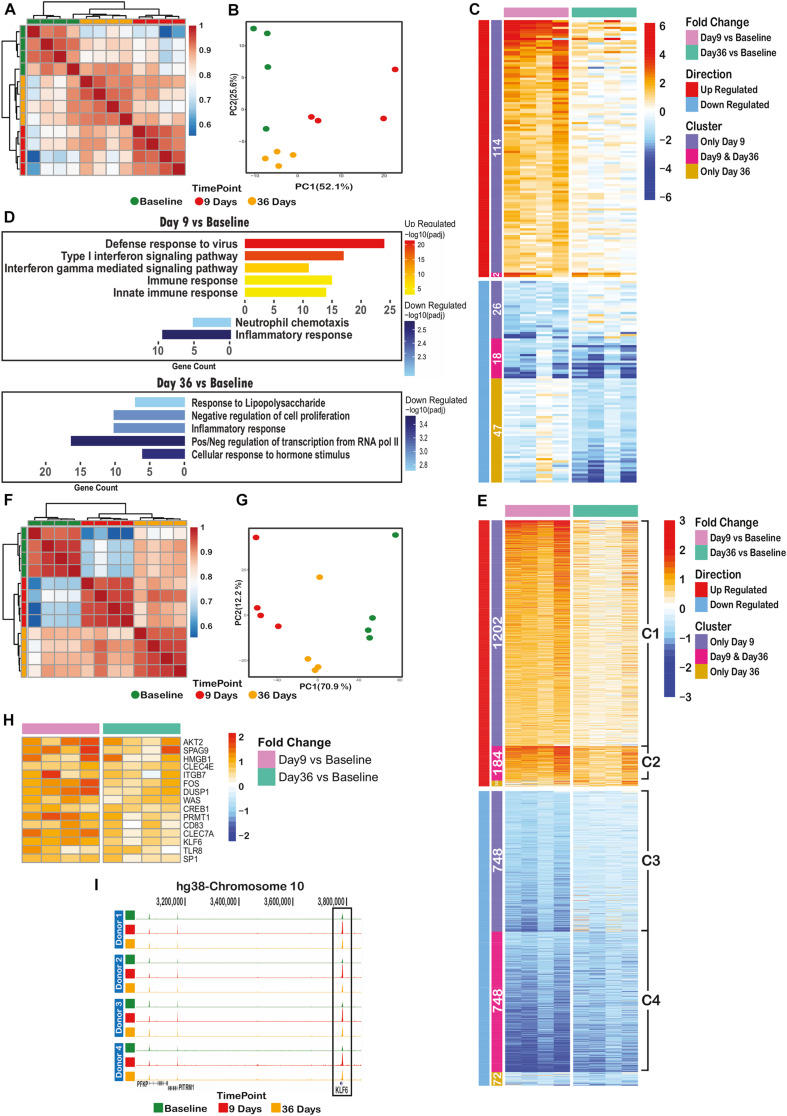
Controlled Human Malaria Infection has long lasting transcriptomic and epigenomic effects on monocytes. **(A)** Spearman correlation clustering of significant differential expressed (DE) genes between at least one of the time points (207 genes), **(B)** Principal component analysis plot of DE genes over the time course, **(C)** heatmap of the log2(fold change over the baseline) of DE genes, clustered by their behavior over the time course (differentially expressed on only day 9 or only day 36 or both of these time points), **(D)** significant gene ontology terms associated with differentially expressed genes on day 9 (upper panel) or day 36 (lower panel), **(E)** heatmap of log2(fold change over the baseline) of differential ChIP regions, clustered by their behavior over the time course (differential in only day 9 or only day 36 or both of these time points over the baseline H3K4me3 level), **(F)** spearman correlation clustering of significantly dynamic H3K4me3 histone ChIP peaks between at least one of time points (2984 peaks), **(G)** principal component analysis plot of dynamic peaks over the time course, **(H)** heatmap of up-regulated H3K4me3 regions (annotated to their nearest promoter) remained elevated on day 36 with immunogenic functions. **(I)** H3K4me3 histone peak around KLF6 gene promoter, indicating an increase in signal level at day 9, following a decrease on day 36 while still significantly higher than baseline level.

RNA-sequencing (RNA-seq) of CD14+ cells isolated from four volunteers at baseline, day 9 and day 36 post challenge (Figure 1B) identified 207 out of 8796 detectable genes that were significant differentially expressed (*p*_*adj*_ < 0.05 and fold-change > 2) across all time points and volunteers. Heatmap, correlation and principal component analysis of the 207 dynamic genes show a clear distinction between time points, indicating reprogramming of transcription over the time course (Figures 3A–C). Nearly all of the up-regulated genes (116) were maximally expressed at day 9. The majority of these were associated with antiviral responses and the type I interferon signaling pathway (Figure 3D, upper panel). At day 36, several anti-inflammatory genes are significantly down-regulated (*p*_*adj*_ < 0.05 and FC > 2), such as NFKBID, NFKBIZ, DUSP1, 2, and ATF3 (Figure 3D, lower panel) in line with the observed stronger response to a secondary stimulus (Figures 2A–C).

We also performed chromatin immunoprecipitation followed by deep sequencing (ChIP-seq) on histone 3 trimethylation at lysine 4 (H3K4me3), an active mark of promoters, on monocytes from all three timepoints (Figure 1B). Unlike the overall RNA transcription data, the overall H3K4me3 ChIP-seq data showed a clear clustering of time point-specific samples (Supplementary Figure 1). Around 17% (2984/17990) of identified H3K4me3 peaks were dynamic between at least two time points (*p*_*adj*_ < 0.05, fold-change > 1.5). There were 1,386 up- and 1,496 down-regulated peaks between baseline and day 9 time points, while 224 up- and 820 down-regulated regions were identified in the comparison of baseline and day 36 samples (Figure 3E). Spearman correlation and PCA of these dynamic genes revealed clear differences between samples of different time points (Figures 3F,G).

Next, we assessed the extent of retained epigenetic changes over the time course. As illustrated in Figure 3E, around 87% (1202/1386) of regions with elevated H3K4me3 on day 9 (cluster C1) slowly lost their elevated marking over time but did not reach baseline levels at day 36. Similarly, the loss of H3K4me3 at day 9 of infection was not entirely restored to base line level at day 36 (Figure 3E, clusters C3, C4). At a small number of genomic sites (184, cluster C2 in Figure 3E), H3K4me3 levels remained at significantly (*p* = 1.1e-4) higher level as compared to baseline, indicative of epigenetic memory after *P. falciparum* infection.

Importantly, genes with retained elevated H3K4me3 levels at their promoters (cluster C2 in Figure 3E) did not show significantly altered transcription levels at day 36, suggesting that these genes are primed for a better immune response and will only be activated upon a secondary stimulation. In this case, the nearest genes linked to cluster C2 regions are likely to contain inflammatory response related genes. We therefore inspected the list of 184 regions (cluster C2) and found 15 known immune-related genes with elevated H3K4me3 on day 36, reminiscent of primed promoters (Figure 3H). As an example, Figure 3I shows the H3K4me3 peaks at the KLF6 promoter that is elevated on day 9 and decreased to the level observed on day 36, which is, however, still significantly higher than baseline level.

Taken together, this indicates that *P. falciparum* infection induces a marked increase in monocyte pro-inflammatory cytokine production to heterologous stimuli that is associated with epigenomic changes in monocytes that are maintained for at least several weeks.

## Discussion

In the present study, we detect a marked increase in monocyte pro-inflammatory cytokine production to heterologous stimuli 5 weeks after a Controlled Human Malaria Infection in previously malaria-naïve volunteers. This pattern is similar to trained immunity seen in humans after BCG vaccination (Kleinnijenhuis et al., 2012; Arts et al., 2018), and represents the first observation of innate immune memory after infection with a human pathogen. These findings corroborate and extend an earlier study that found increased monocyte cytokine responses after *in vitro* exposure to *Plasmodium falciparum* (Schrum et al., 2018). Here, *P. falciparum* exposure and subsequent monocyte training occurs *in vivo* after a low density infection, transmitted through the natural, mosquito-borne route. As the study design allows for longitudinal sampling of individuals at multiple time points before and after infection, CHMI presents an ideal opportunity to study the induction of trained immunity in humans.

Studies comparing *in vitro* LPS tolerance and β-glucan training have suggested that these stimuli induce opposing functional programs (Foster et al., 2007; Ifrim et al., 2014; Saeed et al., 2014; Novakovic et al., 2016). In the present study the changes in monocyte function are biphasic, with a transient tolerance during infection followed by stronger pro-inflammatory response after parasite clearance. The responses observed after malaria infection are reminiscent of the effect of LPS, which can lead to hyper-responsiveness or tolerance depending on dose and timing (Salomao et al., 2012; Ifrim et al., 2014). It would be interesting to determine monocyte responses at additional time points in future studies, in order to determine the precise course of these changes.

A recent study by Dobbs et al. (2017) determined monocyte TLR responses in Kenyan children with acute malaria. In contrast to our findings, they found similar cytokine production during the infection and 6 weeks after treatment, which were both higher than in North American adults. However, these children may have had repeated (asymptomatic) parasitemia between the time points. Further studies are needed to determine whether natural malaria infections with high and/or prolonged parasitemia also induce trained immunity.

In agreement with the cytokine assays, we observed a genome-wide reprogramming of monocytes in both transcription (RNA-seq) and epigenome (ChIP-seq) levels. Our RNA-seq analysis shows that several inhibitors of the NF-kB and MAPK signaling pathways were significantly down-regulated 36 days after infection, including NFKBID, NFKBIZ, DUSP-1, -2 (Dauletbaev et al., 2011), NR4A2 (Crean et al., 2015), and tristetraprolin (TTP or ZFP36). It has been demonstrated that TTP targets the AU-rich region (ARE) of TNF-α and IL-6 resulting in mRNA degradation of these genes (Tiedje et al., 2016). Down-regulation of ZFP36 suggests a potential mechanism for the observed priming of monocytes toward a better response against secondary stimuli at this time point.

Among the 15 genes with increased H3K4me3 at day 36 in the C2 cluster, several are of special interest to the induction of trained immunity. AKT2 is a member of the PI3K/AKT/MTOR pathway that is important in the induction of immunometabolic changes in trained immunity (Cheng et al., 2014). PRMT1 is an epigenetic enzyme that induces histone methylation, and HMGB1 is an important chromatin protein that regulates transcription and is a mediator of inflammation. Expression of the CLEC4E and CLEC7A receptors was also increased, which is interesting as CLEC7A (also known as dectin-1) is the receptor for the induction of trained immunity by β-glucan (Ifrim et al., 2013).

Unexpectedly, several inflammatory response genes such as IL1b, IL8 and CCL3 were down regulated at day 9 and 36 (Figure 3C). A plausible explanation of the observed down-regulation of inflammatory response at day 9 is that the initial cytokine response to the infection has ended before day 9 despite the clear parasitemia phenotype at this time point. Contrary to the results from Schrum et al. (2018), we could not identify an increase in H3K4me3 peaks over the promoter region of TNFA on day 9, which may reflect differences between *in vitro* and *in vivo P. falciparum* exposure.

This observation of reduced monocyte responsiveness on day 9 is in stark contrast to the increase in pro-inflammatory responses in total PBMCs frequently seen in malaria (McCall et al., 2007; Hartgers et al., 2008; Franklin et al., 2009; Ataide et al., 2014), including in our CHMI model (McCall et al., 2007). This suggests that the reduced cytokine production we observe is monocyte specific and/or can be overcome during the setting of active infection by the presence of and/or interaction with lymphocytes. Indeed, a recent study examined the interplay of T cells and tissue macrophages during infection, and demonstrated the importance of lymphocytes in boosting the induction of trained immunity (Yao et al., 2018).

This study was limited by its small sample size, however, most changes occurred in all the 5 volunteers investigated, and are consistent across multiple stimuli and cytokines.

To date only a few pathogens have been shown to induce trained immunity. As such, our observation of innate memory after a human parasitic disease is significant, and it suggests that trained immunity occurs after the regular encounters with pathogens. As we also recently showed that BCG-induced trained immunity affects the immmune response against malaria infection (Walk et al., 2019), further study is warranted to determine whether trained immunity is induced in natural malaria or after attenuated sporozoite immunization, and whether this contributes to protection against repeated infections.

## Methods

### Clinical Trial

Five healthy, malaria-naive male and female volunteers were screened as described previously (Roestenberg et al., 2009) and enrolled as controls in an open-label vaccine trial (registered under NCT02080026). The trial was approved by the Central Committee on Research Involving Human Subjects (CCMO NL48301.091.14) of the Netherlands. Volunteers were infected at the Radboud university medical center (Nijmegen, The Netherlands) in May 2015 by bites from 5 *P. falciparum* NF54-strain infected *Anopheles stephensi* mosquitoes according to established protocols (Walk et al., 2017). Parasitemia was assessed by prospective, daily qPCR from day 6 until day 21 post infection, and treatment was initiated when parasitemia reached 100 parasites/milliliter, as reported previously (Walk et al., 2016).

### Isolation and Stimulation of PBMCs and Monocytes

Blood was collected in ethylene-diamine-tetra-acetic acid (EDTA) vacutainers (Becton Dickinson). Peripheral blood mononuclear cell and monocyte isolation and stimulation was performed as described previously (Kleinnijenhuis et al., 2012). In short, PBMCs were separated using Ficoll-Paque (GE healthcare, United Kingdom) density centrifugation and monocytes were isolated using CD14+ selection magnetic separation (MACS; Miltenyi Biotech). For culture, cells were suspended in RPMI (Roswell Park Memorial Institute medium; Invitrogen) supplemented with 50 μg/mL gentamicin (Centrafarm), 2 mM Glutamax (GIBCO), and 1 mM pyruvate (GIBCO). PBMCs were cultured at 2.5 × 10^6^/mL and monocytes at 0.25 × 10^6^/mL in a final volume of 200μL/well in a 96-well plate. Cells were stimulated with RPMI, sonicated *Mycobacterium tuberculosis* H37Rv (5 μg/mL), heat-killed *Candida albicans* (1 × 10^6^/mL, strain UC820) or *Staphylococcus aureus* (1 × 10^6^/mL clinical isolate), *Salmonella typhymurium* (1 × 10^6^/mL, clinical isolate) or *Escherichia coli* LPS (10 ng/mL, serotype 055:B5, Sigma-Aldrich) for 24 h.

### *In vitro* Monocyte Training

Cells were isolated from buffy coats from healthy adult donors (*n* = 6), obtained from Sanquin Bloodbank (Nijmegen, The Netherlands). Experiments were performed using previously published protocols (Novakovic et al., 2016). In short, from total PBMCs, monocytes were isolated using a high-density hyper-osmotic Percoll gradient. Monocytes were seeded at 0.1 × 10^6^ cells/well in flat-bottom 96-well plates and allowed to adhere for 1 h. Non-adherent cells were washed away with PBS, and monocytes were incubated with schizont and trophozoïte-stage *P. falciparum* NF54-strain-infected red blood cells or uninfected red blood cells for 24 h. After washing, monocytes were allowed to rest in medium (see above) for 5 days. After 5 days they were restimulated with *Escherichia coli* LPS (10 ng/mL, serotype 055:B5, Sigma-Aldrich) for 24 h.

### Cytokine Measurements

Cytokine measurements were performed in the supernatants collected after 24 h or 7 days using commercial ELISA kits from R&D Systems (TNF-α, IL-1β, IL-10, IL-17, and IL-22; Minneapolis, MN, United States) or Sanquin (IL-6, IFN-γ; Amsterdam, the Netherlands), as previously described (Kleinnijenhuis et al., 2012).

### Leukocyte Differentiation Counts

Absolute lymphocyte and monocyte counts were determined directly in EDTA whole blood by the Department of Laboratory Medicine of the Radboud university medical center (Nijmegen, Netherlands), using a Sysmex XE-5000-14 analyzer.

### Statistical Analysis

Analyses were performed in GraphPad Prism 5.03 for Windows.

### Total RNA Extraction and cDNA Synthesis for RNA-Seq

Total RNA was extracted from CD14+ monocytes using the QIAGEN RNeasy RNA extraction kit (QIAGEN, Netherlands) and on-column DNaseI treatment. Utilizing riboZero rRNA removal kit (Illumina) ribosomal RNA was removed from total RNA. RNA was then fragmented into approximately 200 bp fragments by incubation for 3 min at 95C in fragmentation buffer [200 mM Tris-acetate, 500 mM Potassium Acetate, 150 mM Magnesium Acetate (pH 8.2)]. First strand cDNA synthesis was performed using SuperScript III (Life Technologies), followed by synthesis of the second cDNA strand, recruiting DNA polymerase and DNA ligase (NEB). Libraries for RNA-sequencing were prepared using the KAPA hyperprep kit (KAPA Biosystems), according to the manufacturer’s protocol.

### Chromatin Immunoprecipitation

CD14+ monocytes were fixed with 1% formaldehyde (Sigma) at a concentration of approximately 10 million cells per ml. Fixed cell preparations were sonicated for 7 cycles (30 s on, 30 s off) using a Diagenode Bioruptor Pico sonicator. Around 1 million cells were mixed with dilution buffer, protease inhibitor cocktail (Roche) and 1 mg of H3K4me3 antibody (Diagenode) and incubated overnight at 4C with rotation. Pre-washed protein A/G magnetic beads mixture, diluted in 0.15% SDS and 0.1% BSA, added to the chromatin/antibody mix and incubated for 60 min at 4C. Beads were washed with 400μl wash buffer for 5 min at 4C (with rotation). Five rounds of washes were done. After wash, chromatin was eluted using elution buffer. Eluted chromatin mixed with 8 μl 5M NaCl, 3μl proteinase K, and samples were incubated for 4 h at 65C with rotation. Decrosslinked samples were purified using Qiaquick MinElute PCR purification Kit (Qiagen) and eluted in 20 μl EB. Purified immunoprecipitated chromatin was used for library preparation.

### Library Preparation for Next Generation Sequencing

Illumina libraries for next generation sequencing was done using the Kapa Hyper Prep Kit (Roche), according to the manufacturer’s protocol. Briefly, for end repair and A-tailing double stranded DNA, was incubated with end repair and A-tailing buffer and enzyme for 30 min at 20C and then for 30 min at 65C. Subsequently, adapters were ligated using DNA ligase, and Illumina NextFlex adapters, specific for each sample, for 15 min at 20C. Post-ligation cleanup was performed using Agencourt AMPure XP reagent and adapter-ligated samples were eluted in 20 ml elution buffer. Libraries were amplified by mixing 2x KAPA HiFi Hotstart ReadyMix and 10x Illumina library amplification primer mix and using PCR for 10 cycles. Amplified PCR products were purified using the QIAquick MinElute PCR purification kit (Qiagen). Utilizing E-gel, 300 bp fragments selected from each sample. Correct size selection was approved by BioAnalyzer analysis. Sequencing experiments were performed in Illumina NextSeq 500 machine in the paired end fashion.

### RNA-Seq Data Analysis

To infer gene expression levels, RNA-seq reads were aligned to the hg38 human reference transcriptome using STAR (Dobin et al., 2013). Genes with fewer than 50 reads on average in all time points (baseline, day9 and day36) were excluded from downstream analysis. Utilizing DESeq2 libraries normalized and differentially expressed genes determined (Love et al., 2014). Genes with more than twofold change between at least two time points, who have the same up- or down-ward trend in all donors, were identified as dynamic genes. In addition, genes with more than twofold change difference between at least two time points and *p*_*adj*_ value < 0.05 were designated as significant differentially expressed genes.

### ChIP-Seq Data Analysis

Reads from NGS results were mapped to hg38 human reference genome using bowite2 (Langmead and Salzberg, 2012). Duplicated fragments were excluded from further analyses steps. Utilizing MACS2 software ChIP peaks were called on remaining reads with the *p-*value of 1e-8 (Zhang et al., 2008). Called peaks across all samples were merged and reads aligning within merged peaks from each sample were counted using peakstats. Quantified ChIP-seq data were normalized using DESeq2. Peaks with more than 1.5 fold change and *p*_*adj*_ < 0.05 between at least two of time points were identified as significant differential peaks. For the intersection analysis of ChIP and RNA-seq data, H3K4me3 peaks near the promoter regions of differentially expressed gene were extracted.

## Data Availability Statement

The processed normalized RNA and ChIP-seq data set has been submitted to Gene Expression Omnibus (GEO) and can be accessed using accession number GSE137044.

## Ethics Statement

Five healthy, malaria-naive male and female volunteers were screened as described previously (Roestenberg et al., 2009) and enrolled as controls in an open-label vaccine trial (registered under NCT02080026). The trial was approved by the Central Committee on Research Involving Human Subjects (CCMO NL48301.091.14) of the Netherlands. The patients/participants provided their written informed consent to participate in this study.

## Author Contributions

JW, FK, LB, MN, HS, and RS contributed to study and experiment design. JW, FK, LB, RA, and BB performed the experiments. JW and FK performed data analysis. JW, FK, MN, HS, and RS wrote the manuscript. All of the authors contributed intellectual content, fulfill the criteria for authorship, and read and approved the final manuscript.

## Conflict of Interest

The authors declare that the research was conducted in the absence of any commercial or financial relationships that could be construed as a potential conflict of interest.

## Supplementary Material

Supplementary Figure 1 Spearman correlation of RNA-seq and H3K4me3 ChIP-seq data. Spearman correlation of overall RNA-seq data (8,796 genes) (left), along with spearman correlation clustering of overall H3K4me3 ChIP-seq data (right). Overall ChIP-seq peaks (17,990 peaks) show a clear clustering of samples from each time point, indicating the epigenome basis of trained immunity phenotype.

Supplementary Table 1 Characteristics of CHMI volunteers.

Supplementary Table 2 List of Differentially Expressed Genes across RNA-seq Samples.

Supplementary Table 3 List of Differential H3K4me3 Regions across ChIP-seq Samples.
