# Supplementary figures and images for: Controlled Human Malaria Infection Induces Long-Term Functional Changes in Monocytes
Source: Front Mol Biosci. 2020 Nov 26;7:604553. doi: 10.3389/fmolb.2020.604553 (PMC7726436; doi:10.3389/fmolb.2020.604553)

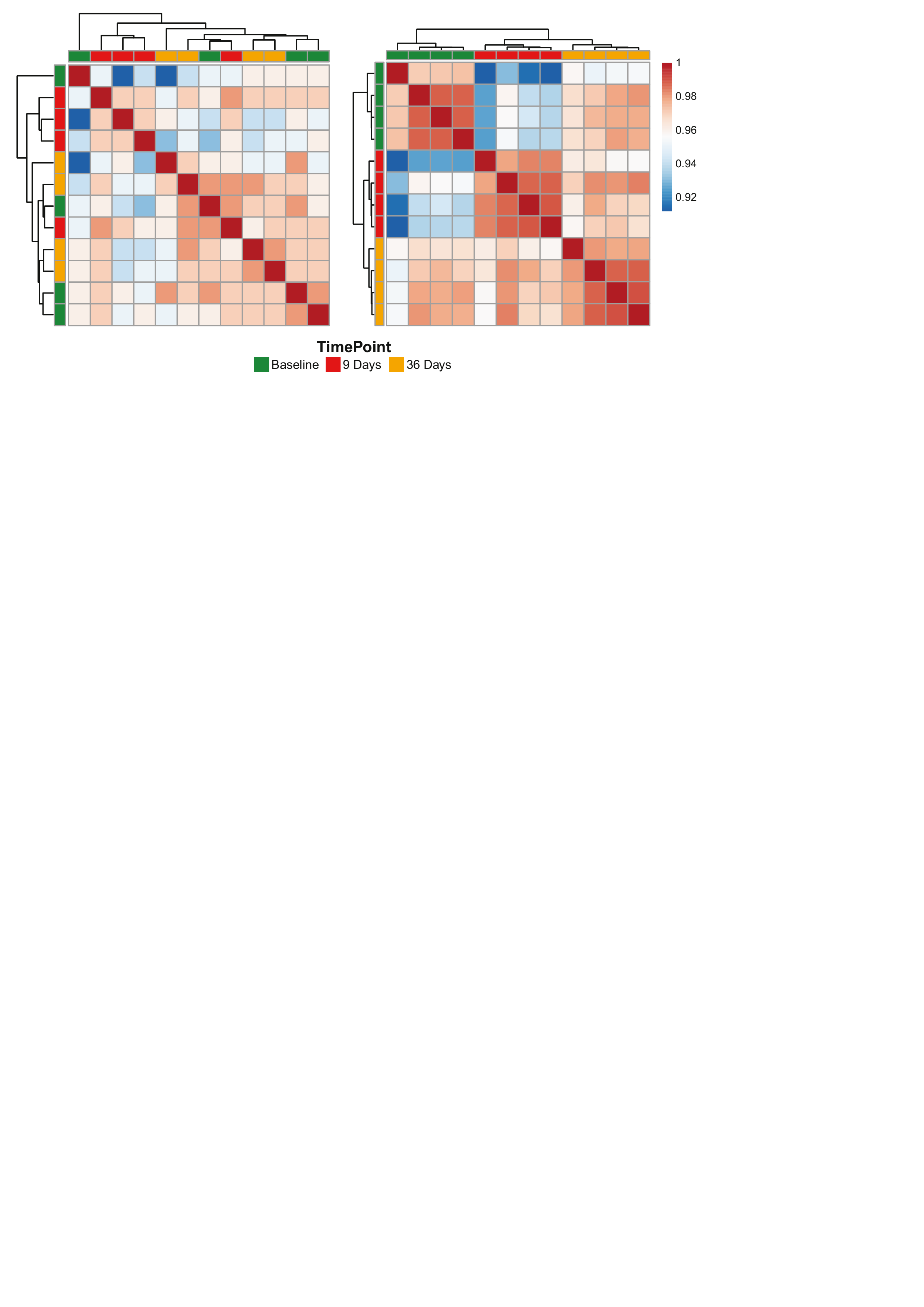

Supplement: Supplementary Figure 1 — Spearman correlation of RNA-seq and H3K4me3 ChIP-seq data. Spearman correlation of overall RNA-seq data (8,796 genes) (left), along with spearman correlation clustering of overall H3K4me3 ChIP-seq data (right). Overall ChIP-seq peaks (17,990 peaks) show a clear clustering of samples from each time point, indicating the epigenome basis of trained immunity phenotype. [file Image_1.TIF]
